# Assessment of post-infarct ventricular septal defects through 3D printing and statistical shape analysis

**DOI:** 10.2217/3dp-2022-0012

**Published:** 2023-01-18

**Authors:** Ashar Asif, Andrew IU Shearn, Mark S Turner, Maria V Ordoñez, Froso Sophocleous, Ana Mendez-Santos, Israel Valverde, Gianni D Angelini, Massimo Caputo, Mark CK Hamilton, Giovanni Biglino

**Affiliations:** 1 Bristol Medical School, University of Bristol, Bristol Royal Infirmary, Upper Maudlin St, Bristol, BS2 8HW, UK; 2 Bristol Heart Institute, Bristol Royal Infirmary, Upper Maudlin St, Bristol, BS2 8HW, UK; 3 Pediatric Cardiology Unit, Hospital Virgen del Rocio and Institute of Biomedicine of Seville (IBIS), Seville, E-41013, Spain; 4 School of Biomedical Engineering and Imaging Sciences, King’s College London, King’s Health Partners, St Thomas’ Hospital, SE1 7EH, UK; 5 Department of Clinical Radiology, Bristol Royal Infirmary, Upper Maudlin St, Bristol, BS2 8HW, UK; 6 National Heart and Lung Institute, Guy Scadding Building, Imperial College London, London, SW3 6LY, UK

**Keywords:** 3D printing, myocardial infarction, statistical shape modeling, ventricular septal defect

## Abstract

**Background:**

Post-infarct ventricular septal defect (PIVSD) is a serious complication of myocardial infarction. We evaluated 3D-printing models in PIVSD clinical assessment and the feasibility of statistical shape modeling for morphological analysis of the defects.

**Methods:**

Models (n = 15) reconstructed from computed tomography data were evaluated by clinicians (n = 8). Statistical shape modeling was performed on 3D meshes to calculate the mean morphological configuration of the defects.

**Results:**

Clinicians’ evaluation highlighted the models’ utility in displaying defects for interventional/surgical planning, education/training and device development. However, models lack dynamic representation. Morphological analysis was feasible and revealed oval-shaped (n = 12) and complex channel-like (n = 3) defects.

**Conclusion:**

3D-PIVSD models can complement imaging data for teaching and procedural planning. Statistical shape modeling is feasible in this scenario.

## Plain language summary

Following heart attacks, the heart muscle becomes scarred and weaker, making it prone to tearing under high pressures. These tears are known as ‘post-infarct ventricular septal defects’. Their shape varies greatly as the heart beats. The approach to fixing these can range from plugging them with a device or patching them by open heart surgery. We created 15 3D-printed models of hearts with these kinds of defects and made digital reconstructions of the tears to see the different sizes/shapes that they can have. Doctors agreed that 3D-printed models could help in planning repairs and training other doctors. The digital reconstructions of the tears showed that many were round, but some had irregular shapes which would mean devices used to fix them may not fit.

## Tweetable abstract

Case series of 3D-printed heart models with post-infarct ventricular septal defects. These 3D models can be useful for teaching or device procedure planning and could be used to complement dynamic data of the defect.

Post-infarct ventricular septal defect (PIVSD) is a rare but serious complication of myocardial infarction (MI) developing from a total occlusion of either the left or right coronary artery typically 3–5 days after the infarct. It has an incidence rate of <1% but a mortality rate of 90% within 2 months if left without closure and 53% at 1 year if surgically repaired [1,2]. Repair is approached with a left-sided ventriculotomy [3,4]. Optimal outcomes from surgical repair are seen if the procedure is delayed some time (operative mortality rates of 54.1% if repair occurs ≤7 days from MI and 18.4% if >7 days from MI [5]), giving time for oedema to reduce and the infarcted area to develop a firmer surface for sutures to anchor upon [6]. Percutaneous occlusion via a transcatheter approach has also been demonstrated to be a viable management option, especially in PIVSDs <15 mm, albeit with a risk of device embolization [7,8]. Devices such as the AMPLATZER™ Post-infarct Muscular VSD Occluder have shown 61% event-free survival at 5 years [9]. Like surgical closure, delayed percutaneous occlusion has been shown to be of benefit to patient outcomes [10]. Delayed intervention in these patients allows for a window for joint surgical and cardiology meetings to plan management options.

Unlike congenital ventricular septal defects (VSDs), PIVSDs have been shown to vary in morphology from *in vivo* computed tomography (CT) and MRI analysis [11], making predesigned devices for transcatheter closure inappropriate. The use of computer-aided design and 3D-printing technology has been shown to be a useful tool in conjunction with imaging modalities in anatomical analysis and preinterventional planning in several cardiac pathologies [12–16]. Additionally, techniques such as statistical shape modeling are showing promise to quantify morphological variability in a population of cardiac or vascular shapes [17–21], but to our knowledge this framework has never been applied to septal defects, potentially with highly variable geometries, thus suggesting an additional area of meaningful investigation.

We present here a series of cases which illustrate the feasibility of using 3D-printed models of 15 patients with PIVSD; we aimed to evaluate the usefulness of such models in clinical assessment, communication and intervention planning. As a secondary aim of this study, we assessed the feasibility of statistical shape modeling in quantifying the average morphology of PIVSDs, testing the framework in the same sample.

## Materials & methods

### Case selection

Consecutive cases of PIVSDs presenting to Bristol Heart Institute, UK between 2006 and 2016 were selected as reported in a previous study [11]. PIVSDs were diagnosed by transthoracic echocardiography. There were 15 cases that had available CT images with DICOM^®^ (Digital Imaging and Communications in Medicine) files available for 3D reconstruction.

### CT protocol

The imaging protocol follows that of a previous study and is reported in detail elsewhere [11]. Of the 15 CT examinations acquired locally, five were acquired with 1-mm voxels (Sensation 16, Siemens Medical Solutions, Erlangen, Germany) and ten with 0.6- to 0.75-mm voxels (Definition AS+, Siemens Medical Solutions). These voxel sizes (isotropic) are appropriate to clinically assess defects that are frequently >20 mm in diameter.

### 3D printing

**Figure 1.**
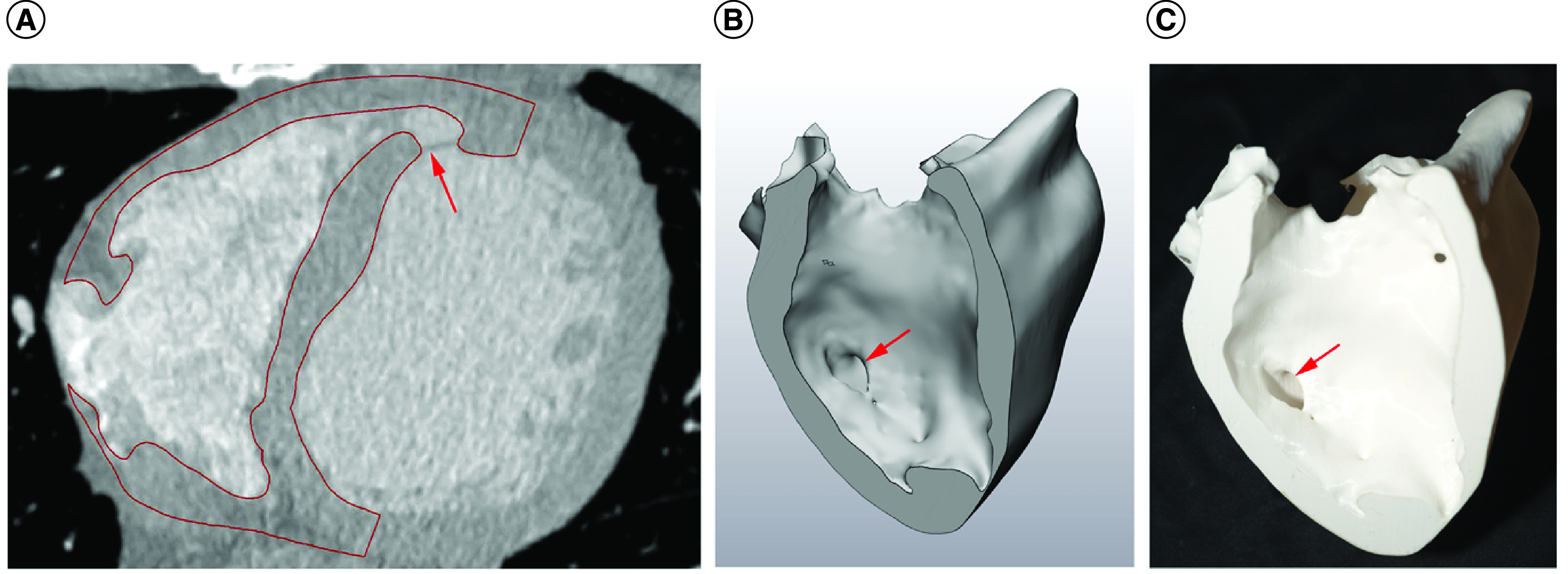
Segmentation of computed tomography to produce the 3D models. **(A)** The computed tomography image is segmented and a mask produced, which is converted to **(B)** a 3D volume. **(C)** This is, in turn, converted into a stereolithography file suitable for printing a final model. The ventricular septal defect is indicated by the arrow.

Reconstruction of the 3D heart models was performed using Mimics Research (Mimics Research 19.0, Materialise, Leuven, Belgium). A %ECG phase greater than 70% was selected due to the heart being in diastole and hence accentuating the defect. 3D-model segmentation was achieved through selecting the appropriate threshold to create two masks for each model of each case: blood pool and whole heart. Values for thresholding were dependent on the original image intensity and attenuation. These were manually selected for each case, and the lower boundary of the threshold was set to capture an adequate area for each mask while aiming to avoid capturing unnecessary structures such as skeletal structures. Each image underwent iterative steps of removing background and adding necessary structures using region regrowing, and multiple slice edit tools were used to produce masks for the blood pool and whole heart. The aforementioned methods have been published elsewhere [13,22]. Using the Boolean subtraction tool, the blood pool and whole-heart 3D reconstruction were subtracted from each other, resulting in a hollowed, isolated myocardium model. These were smoothed, sliced laterally to the left of the interventricular septum to allow direct visualization of the PIVSD as it would be approached surgically (3-matic Research 11.0, Materialise) to cater for both surgical and interventional cardiology stakeholders. The virtual models were exported as stereolithography (STL) files. The models were 1:1 in size and focused on the ventricles. The resulting 3D reconstructions were verified visually by a cardiologist against the original CT dataset. An example of the model production process is shown in Figure 1. The STL files were imported to the 3D-printing software (PreForm 2.10.3, Formlabs, MA, USA) where model orientation and scaffolding were set before printing the model. The models were printed on either a Form 2 SLA printer (Formlabs) or an Ultimaker S5 (Ultimaker, Geldermalsen, The Netherlands) depending on model size, and appropriate resins were used accordingly; that is, models were printed in either white resin (FLPWHO04, Formlabs), or PLA White (1613, Ultimaker, using Natural PVA 9731 as a scaffolding material, according to manufacturer’s instructions). Completed models were then quality-assessed by a biomedical engineer with experience of 3D-printing cardiac models and a cardiac radiologist for accuracy compared with the original CT scans and for model presentability.

### Model evaluation

To evaluate the models, a group of clinicians (three cardiac surgeons, three interventional cardiologists, one cardiologist with expertise in cardiac MRI and one cardiac radiologist) were invited to review the models with the corresponding series of CT images. The clinicians were invited to openly comment on the models based on a series of questions designed to determine the utility of the models in four main areas: understanding the anatomy, use for teaching, use for procedural planning and static versus dynamic models. Their responses were recorded and transcribed. Thematic analysis was then performed to identify dominant themes in the responses [23]. This approach was chosen based on the fact that we wanted to focus on the explanatory dimension and the evaluative dimension, understanding what features of 3D-printed models are relevant in this context, and for this reason a control group was not included as this was not the focus of the study (e.g., comparing 3D model addition vs 3D image alone).

### Statistical shape modeling

As part of the patient-specific modeling approach presented in this study, the shape of the PIVSDs was also studied in isolation. Essentially, while the 3D-printing protocol reconstructed the myocardium highlighting the defect, this complementary approach focused on characterizing the morphology of the defect itself. As such, the area of the VSD was reconstructed following the same principles of image segmentation outlined above (Mimics Research), creating a mask for the VSD itself (Figure 1). The VSD reconstructions were checked by two cardiologists. These were then exported as 3D volumes (STL files) for statistical shape modeling. All reconstructed VSD 3D shapes were aligned (based on their barycenter) and imported into Deformetrica software (www.deformetrica.org) for shape analysis [24]. The algorithm calculates a mean morphological configuration (‘template’) and outputs quantitative information (‘shape vectors’) on the deformation of each individual shape from the template defining dominant shape features (‘shape modes’). As such, the morphological variability of each shape can be characterized in detail and in its three-dimensionality. The detailed methodology of this approach has been reported elsewhere [16–19]. The focus of the analysis here was on the feasibility of firstly, applying the framework to the reconstructed PIVSD geometries; and secondly, extracting the template for the purpose of characterizing the average morphology of the PIVSDs.

## Results

**Figure 2.**
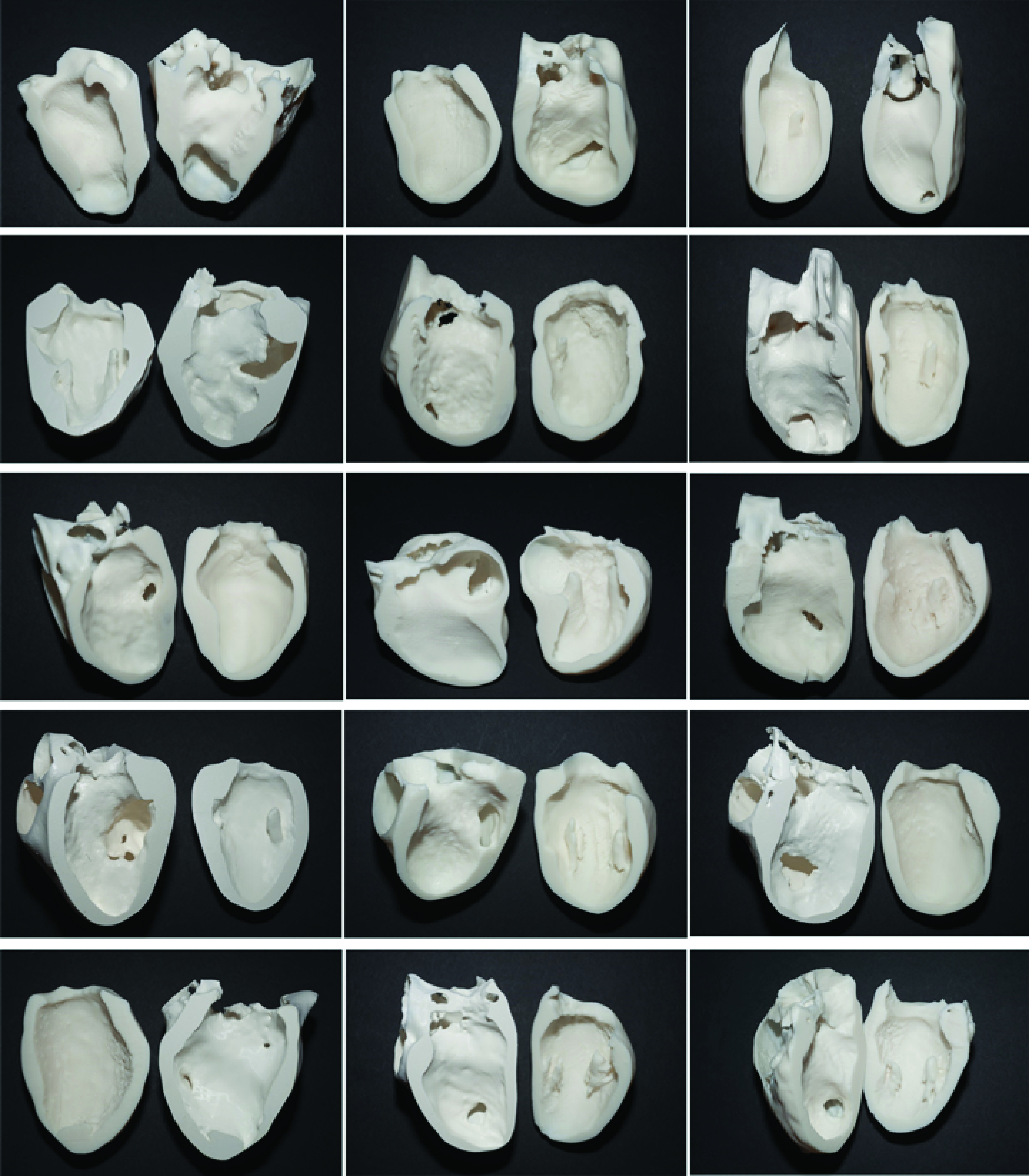
Montage of photographs of each of the 15 3D-printed models. From top left to right: VSD03, VSD05, VSD06, VSD10, VSD13, VSD14, VSD22, VSD23, VSD26, VSD27, VSD29, VSD30, VSD32, VSD34 and VSD35. Arrows indicate post-infarct ventricular septal defects.

All 15 models were successfully printed and are shown in Figure 2. The models were reviewed by eight clinicians (clinician feedback in the Supplementary Material) and the dominant themes emerging from the transcripts of the interviews were:

1. Good understanding of the anatomy: all eight participants said that the models demonstrated the anatomy well, capturing the heterogeneity of the defects. Two clinicians said the models in the series were still complex to assess, and five suggested that the inclusion of anatomical landmarks would be helpful.
2. Value for teaching: all eight participants suggested that the models could be used for teaching, suggesting using the models as part of a training day for trainees.
3. Usefulness for procedural planning: seven of the eight participating clinicians commented that the models would be useful for procedural planning. Other suggestions included using them to test devices prior to implantation or working collaboratively with industry to develop specific devices for treatment of the condition. One surgeon felt that the models would be helpful for an interventionalist but not a surgeon (*“I don’t think it’s very helpful for a surgeon, but definitely this is for planning a device closure because you can, potentially, look at the shape, and maybe change the device shape…”*). There was limited enthusiasm from any of the participants for using the models to explain the procedure to the patient, as they felt patients were unlikely to be well enough.
4. Static versus dynamic models: three of the eight clinicians commented that models do not capture morphological changes occurring during the cardiac cycle, and four suggested that such changes may be better captured using virtual reality. It was also noted by one surgeon that the use of virtual models during online multidisciplinary team meetings, which were increasingly used during the COVID-19 pandemic, could be appealing.

**Figure 3.**
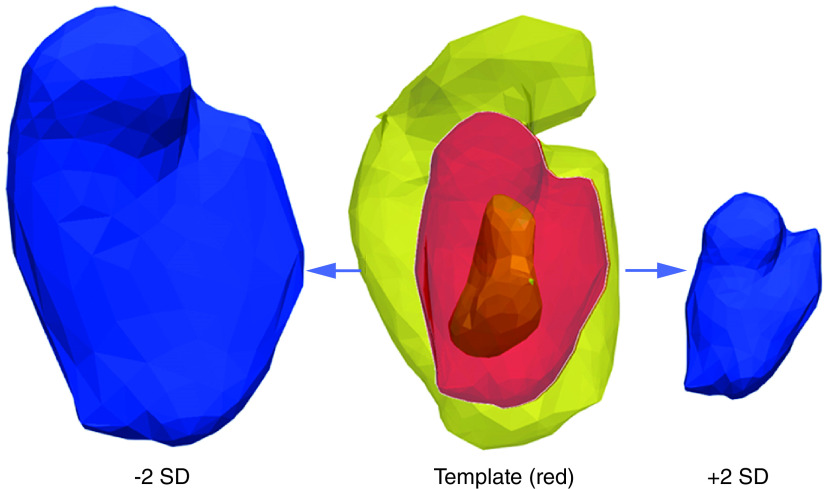
Statistical shape modeling can be used to model the average shape of the defect. Mean configuration of round/oval-shaped septal defects (in red, mean of 12 patients), superimposed with the largest and smallest ventricular septal defect in the sample (in yellow, respectively). In blue, variations around the mean (‘template’), showing ±2 standard deviations (SD).

Finally, statistical shape modeling was feasible. Morphological analysis of the septal defects themselves revealed, unsurprisingly, a great degree of variability among patients. Out of 15 patients, 12 displayed a similar round- or oval-shaped defect of varying size, while three exhibited more complex channel-like shapes. A template was computed for round defects and is shown in Figure 3, highlighting the degree of defect size among these patients and their mean configuration. Mean dimensions were 31 × 21 × 18 mm.

## Discussion

In this study we have demonstrated the feasibility of 3D modeling (including 3D printing) for the study and analysis of PIVSDs. Our case series of 15 patients displayed the intraventricular morphology of PIVSDs and elicited positive responses from interviewed clinicians with respect to displaying the anatomy, training clinicians and preprocedural planning. Additionally, the feasibility of applying a statistical shape-modeling framework to the study of the defects themselves was demonstrated.

3D models of different cardiovascular anatomies and anomalies have previously been produced [13,25,26], and the modeling of VSDs in particular has been shown to have applications in clinical training [27]. A case series of PIVSD models has not been presented before. The cases included in this series ranged in age and complexity.

The 3D models were very well received by the evaluating clinicians, and four dominant themes emerged. They could be used to demonstrate the anatomy of PIVSDs, in particular with a focus on teaching. Given the size of the series and by presenting the models side by side, the heterogeneity of the pathology could be well appreciated. Due to advances in treatment of MI, PIVSDs have become a rare complication [28]; therefore many clinicians will not be familiar with them, and it was suggested that the models could form part of a training day for trainees on PIVSDs. Interestingly, on several occasions, the interviewed clinicians instinctively used the models to demonstrate the anatomy to the interviewer, almost without realizing. The value of 3D-printed models for teaching, including teaching of cardiovascular anatomy, is becoming increasingly recognized [29–32].

It was felt that the models would be of particular use during procedural planning, as we have demonstrated with models in previous cases [33–35]. Of particular interest to the clinicians was the potential ability to use the models to test devices, either prior to implantation or for the development of new devices for the treatment of this pathology. One area where it was felt that these models would not be useful was patient communication. While 3D-printed models of congenital defects have shown potential for improving communication with both young people and their parents [36–38], PIVSDs are usually a very serious complication of MI and, as such, the patient is unlikely to be well enough for a discussion of the procedure.

The limitations of the static 3D models were also noted. The morphology of the PIVSD changes during the cardiac cycle, and this was commented on during the interviews. The model itself is therefore a ‘snapshot’ of one point in time of the PIVSD. For this reason, all models were printed using the end-diastolic phase of the cardiac cycle, when ventricles have higher volumes and the size of the VSD is usually larger. This is key to choose the adequate device size and prevent embolization.

With suitable software, the acquired 4D CT of the PIVSD could be visualized throughout the cardiac cycle, in a virtual environment [39]. An occluder device could then be virtually tested in the PIVSD throughout the cardiac cycle. While this might not be essential for teaching purposes, it is definitely a key feature for device selection, as also discussed in the context of other procedures [15,19,22]. Recently, given that the COVID-19 pandemic has forced many multidisciplinary team meetings online, the virtual model could also represent a suitable medium for presenting and navigating a patient’s anatomy. It should be noted that 3D printing adds an important haptic element (i.e., a 1:1 tangible object) to the insight provided by visualization techniques (3D image, 3D portable document format file etc.) and, as recognized in the literature, 3D printing and imaging have a complementary and meaningful association [40].

Importantly, *in vivo* analysis of PIVSDs has shown that the shape of the defect varies during the cardiac cycle [11], which means that static 3D models inherently present a limitation for assessing size. Arguably, the main purpose of the 3D-printed models would not be sizing of the defect, which would be performed on CT, but they would increase appreciation of the anatomical complexity and spatial orientation, as well as providing visualization of the defect and haptic assessment. This could represent a contribution to the decision-making process (both surgical and interventional), while access to a case series of PIVSDs would hold great educational value, in light of their complexity and rarity.

Sizing of the defect and device selection could also be aided by morphological characterization of the defects, as presented here, by statistical shape modeling. This computational technique, presented as proof of principle and feasibility here, allows the capture of morphological variations in a population and deformations of a predetermined shape. In this context, knowledge of morphological variability could provide useful information not only for device sizing but also for device design, potentially identifying a configuration that would suit a majority of cases. The latter should clearly be explored in a larger case series, but here we have demonstrated the feasibility of the technique. More broadly, the potential of statistical shape modeling is multifaceted. It includes: identifying and quantifying variability in morphology; correlating 3D (not 2D) morphological information with clinical variables using regression techniques; running longitudinal models to observe the progression of changes in morphology (i.e., any potential remodeling); and identifying shapes at higher risk of poor outcomes/complications. The quantitative insight into shape relates to the method’s ability to explore associations between multiple shape features and clinical data, in contrast to a 2D assessment (i.e., a standalone diameter or curvature measure) or a qualitative assessment (i.e., what a shape ‘looks like’). To explore these features of the framework in this population was beyond the scope of the study, and any association or clustering approach would not be meaningful due to the sample size. Nonetheless, the potential of the technique in this clinical scenario warrants future investigation in larger samples of 3D shapes.

From a technical standpoint, models could be manufactured in a compliant or rubber-like material to allow for more realistic representation. Access to different printers and thus materials could allow a user to manufacture flexible models of these anatomies, satisfying individual preferences. If a model were intended to capture the deformation of the defect, careful mechanical characterization of the material would be essential in order to replicate realistic distensibility. Our case series only evaluated 15 cases from a single center, so our study cohort is not necessarily representative of the pathology. Further multicenter trials can help mitigate this limitation and add to our data.

## Conclusion

We have presented a case series of 3D models of PIVSDs and their evaluation by a group of clinicians. It was felt that the models would be most useful in either teaching or device procedure planning, and less so for patient communication. A virtual model would give more dynamic data, but the 3D-printed models could be used to complement this information. We also demonstrated the feasibility of using statistical shape modeling to obtain data on an average PIVSD configuration, which has the potential to inform device design. It is possible, therefore, that the future utility of 3D-printed models and of statistical shape modeling is based around both visualization of the defect and device design. It is likely that it would be possible to use the statistical shape modeling data as a basis for producing a standardized device that could be tailored to a patient. Otherwise, it could be possible to use model data to produce a bespoke device for a particular patient.

**Summary points**

- Post-infarct ventricular septal defect (PIVSD) is a serious complication of myocardial infarction which is associated with poor outcomes even following transcatheter or surgical intervention.
- We evaluated the utility of 3D-printed models in clinical assessment and interventional planning for PIVSDs and performed statistical shape modeling to analyze their morphology.
- Fifteen consecutive patients who underwent electrocardiographically gated computed tomography were included in this study.
- 3D models were produced by reconstruction of the computed tomography images. Models were evaluated by eight clinicians. A thematic analysis identified themes from the model evaluation.
- We carried out statistical shape modeling on the 3D reconstructions to calculate a mean morphological configuration of the PIVSDs and understand characteristics of each morphological variation.
- Feedback highlighted the models’ utility for displaying defects and their potential for interventional and surgical planning, education, training and device development. However, they lacked dynamic representation of the defect.
- Morphological analysis showed 12 PIVSDs had a similar oval-shaped defect, and three had complex channel-like shapes. Mean defect dimensions were 31 × 21 × 18 mm.
- 3D-printed models may be useful for teaching or in device procedure planning and could be used to complement dynamic data of the defect.
- We demonstrated the feasibility of using statistical shape modeling, with promise both for defect sizing and subsequent device selection and for potential device design.
