# Supplementary material for: Assessment of post-infarct ventricular septal defects through 3D printing and statistical shape analysis
Source: J 3D Print Med. 2023 Jan 18;7(1):3DP3. doi: 10.2217/3dp-2022-0012 (PMC9990116; doi:10.2217/3dp-2022-0012)
Supplement: Supplementary file 1 [file 3dp-07-03-s1.docx]

**APPENDIX A**

**Table 1: Examples of feedback from clinicians in relation to dominant themes from analysis of model evaluation.**

| **Theme** | **Positive features** | **Negative features** | **Potential for improvement** |
| --- | --- | --- | --- |
| Understanding of the anatomy | Visualisation (“You see it’s punched out and it’s well defined”; “The visualisation, to me, is stunning. It’s just amazing”; “I think this is anatomy with a model that’s very easy to grasp quickly”).  Capturing anatomical variability (“The first four I’ve looked at are all completely heterogeneous. They’re all in completely different places…”) | Accuracy in including trabeculations (“I’m wondering if what’s happened here is, when this has been printed, it’s incorporated the fine trabeculations into the wall?”) | Including landmarks (“The AV valves are very important”. “…The moderator band and the septal attachments are very important to the relevance of the defect, because it might interfere with your attempt to close them…”) |
| Use for teaching | Displaying complex anatomies to students and trainees (“I think from an educational perspective, […] this is a great series”) |  | Planning specific training event (“We could do a cardiology/radiology/cardiac surgery training day in PIVSDs”). |
| Use for procedural planning | Aiding in evaluating procedural approach (“…this is making me think that crossing from the left is correct, because you’ve got a smoothish left ventricle…”) | Limited use for explaining the procedure to the patients (“They are unlikely to be well enough [in an emergency setting]”) | Liaising with industry to test devices (“Can I put some devices in these [models]?”) |
| Static vs dynamic models |  | Models are reconstructed from diastolic configuration (“…it’s a static model and in reality a VSD is not static – it opens and closes, because the muscle opens, then it contracts and it closes”) | Virtual models could complement the use of printed models ( “…generally in MDTs we use 3D imaging you can spin around on the computer. What’s more, now all the MDTs are remote. It’s not as useful, maybe? But I think from a planning point of view, what device, how big a device and so on, that would be useful.”) |
